# An organoid-guided roadmap for precision delivery of epigallocatechin gallate in oral submucous fibrosis

**DOI:** 10.3389/fbioe.2026.1792350

**Published:** 2026-03-23

**Authors:** Chenpeng Chen, An Gan, Chenggong Huang, Yishun Weng, Tao Wang

**Affiliations:** Hainan General Hospital, Hainan Affiliated Hospital of Hainan Medical University, Haikou, Hainan, China

**Keywords:** epigallocatechin gallate, mucoadhesive delivery, nanomedicine, oral submucous fibrosis, organoids

## Abstract

Oral submucous fibrosis is a chronic, exposure linked mucosal scarring disorder that restricts mouth opening and increases cancer risk. Epigallocatechin gallate offers convergent antifibrotic actions across TGF-β signalling, oxidative stress, inflammation and collagen remodelling, yet clinical effects remain inconsistent because instability and rapid clearance prevent sustained intralesional exposure. This Review proposes a precision delivery roadmap that couples engineered local dosage forms and nanocarriers with patient-derived organoids as a functional translational layer. We describe how organoids can benchmark retention, penetration and spatial drug gradients while quantifying fibrosis aligned endpoints including collagen content, α-SMA, LOX activity, hypoxia and mechanotransduction readouts. Biomarker guided comparisons across molecular subtypes enable responder enrichment and reduce signal dilution in trials. We further outline an auditable evidence chain that connects material attributes to local exposure, mechanism aligned efficacy readouts, safety, and practical constraints such as manufacturability and deployability for clinical adoption. Standardized decision criteria and cross center harmonization are essential for reproducible organoid guided screening.

## 1 Introduction

### 1.1 Epidemiological patterns and the landscape of hazardous exposures

**FIGURE 1:**
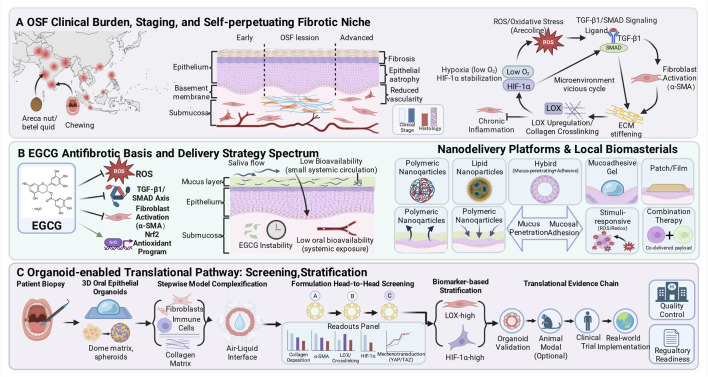
A Schematic overview of EGCG antifibrotic delivery strategies and an organoid-enabled translational pathway in oral submucous fibrosis. A The clinical burden and staging context of oral submucous fibrosis are illustrated by its geographic clustering in areca nut and betel quid chewing populations, together with a schematic trajectory from early to advanced mucosal remodeling, highlighting epithelial atrophy, submucosal collagen accumulation, and reduced vascularity, while situating these changes within a self reinforcing niche in which oxidative stress and TGF-β1/SMAD signaling promote fibroblast activation, LOX mediated collagen crosslinking and matrix stiffening, hypoxia associated HIF-1α stabilization, and chronic inflammation that collectively amplify a vicious microenvironmental cycle; B Driven by EGCG’s multi target antifibrotic activity, including attenuation of oxidative stress and the TGF-β1/SMAD axis, suppression of fibroblast activation, and induction of Nrf2 dependent antioxidant programs, the delivery spectrum is framed around key oral barriers such as the mucus layer, salivary washout, chemical instability, and low systemic bioavailability, and is mapped to nanodelivery and local biomaterial options encompassing polymeric and lipid nanoparticles, hybrid systems integrating mucus penetration with mucosal adhesion, mucoadhesive gels, and patch or film dosage forms, with extensions toward ROS or redox responsive designs and combination therapy for co delivered payloads; C An organoid-enabled translational pathway comprising biopsy-derived three-dimensional oral epithelial organoids, stepwise model complexification via fibroblasts, immune components, collagen matrix and air-liquid-interface culture, head to head formulation screening with a readout panel spanning collagen deposition, α-SMA, LOX associated crosslinking, HIF-1α and mechanotransduction markers, biomarker based stratification including LOX high and HIF-1α high phenotypes, and an auditable evidence chain from organoid validation through optional animal modeling to clinical trial implementation and real world deployment under quality control and regulatory readiness frameworks. Here, the Readouts Panel is intended as a standardized decision gate with predefined effect size cutoffs, for example, at least 30% reduction in collagen deposition and at least 25% reduction in alpha smooth muscle actin signal relative to the fibrotic control, together with a basic safety check on epithelial viability (Created in https://BioRender.com).

Oral submucous fibrosis (OSF) shows marked geographic clustering, closely aligned with high risk exposures such as areca nut chewing (Tilakaratne et al., 2016; Wang et al., 2024). Global burden: approximately 5 million cases worldwide, concentrated in South and Southeast Asia, with India reporting the highest prevalence at about 4% in pooled estimates (Wang et al., 2024; Shen et al., 2020; Shih et al., 2019). Areca nut containing products are recognized as the principal etiologic exposure, and OSF risk increases in a clear dose response relationship with chewing frequency and duration (Sinor et al., 1990; Betel-quid and areca-nut chewing and some areca-nut derived nitrosamines, 2004; Murti et al., 1995). Higher per episode areca nut payload, typical of commercially processed preparations, may accelerate OSF onset relative to traditional homemade quid. In heavily exposed populations, both cumulative incidence and the rate of disease progression rise with greater intensity and longer years of use (Babu et al., 1996; Jain et al., 2017). Risk assessment should therefore extend beyond a binary exposure history to incorporate cumulative dose and post cessation trajectories, because available evidence indicates that fibrotic activity can persist after quitting and may remain partly irreversible, highlighting the dynamic nature of risk (Chen et al., 2023; Auluck et al., 2008). This epidemiological reality constrains future delivery strategies, as real world adherence fluctuates and relapse to areca nut use or coexisting chronic oral inflammation is common, together shaping the reproducibility of treatment benefit (Wollina et al., 2015; Athukorala et al., 2021; Samartzi et al., 2021). Put differently, even robust pharmacology must be coupled to behavioral determinants to achieve stable, population level efficacy. Because OSF is most prevalent in low- and middle-income regions, organoid biobanking strategies must be explicitly cost-efficient and operationally feasible. Recent head and neck organoid biobank work demonstrates that small biopsies can yield expandable patient-derived organoids suitable for long-term banking and standardized functional assays (Issing et al., 2025). To reduce per-sample culture costs, conditioned-media based niche factor delivery can replace multiple recombinant growth factors while maintaining reproducible activity across batches and sites (VanDussen et al., 2019). Operational feasibility in low-resource settings can be further strengthened via a hub-and-spoke workflow (local standardized sampling/cryopreservation, regional expansion and QC) together with autonomy-respecting governance models for organoid biobanks (Lewis and Holm, 2022) (Figure 1).

### 1.2 Translational relevance of clinical staging and histopathological features

With the exposure landscape defined, attention should shift to clinical staging and histopathological grading, because the extent of submucosal fibrosis, collagen deposition, and epithelial alterations varies across disease phases (Rathore et al., 2017). Classically, OSF is stratified into early, intermediate, and advanced clinical stages based on the degree of mouth opening limitation and the palpability or extent of fibrotic bands (Motgi et al., 2021; Khanna and Andrade, 1995). In general, more advanced clinical stages are associated with denser submucosal collagen accumulation, greater epithelial atrophy, and a more pronounced loss of tissue elasticity (Khanna and Andrade, 1995; Utsunomiya et al., 2005; Rajendran, 1994). However, clinical staging does not map perfectly onto histological severity: evidence suggests that some patients with mild clinical manifestations already exhibit substantial fibrotic remodeling on biopsy, whereas others show the opposite pattern (Modak et al., 2015; Ara et al., 2013). For example, biopsies from a subset of intermediate stage cases reveal marked collagen crosslinking and robust fibroblast activation, while residual submucosal tissue from occasional advanced stage cases still shows inflammatory cell infiltration (Trivedy et al., 1999; Ma et al., 1995; Tilakaratne et al., 2006; Gandhi and Prasad, 2017). This imperfect concordance argues against relying on a single scale for response assessment and supports multidimensional evaluation in translational studies.

To increase evidentiary granularity, translational studies should integrate both histological and functional endpoints, capturing objective changes in fibrotic tissue while also quantifying clinically meaningful improvements in mouth opening, burning sensation, and related symptoms (Jones et al., 2024; Gondivkar et al., 2023; More et al., 2020). Accordingly, the design and validation of delivery platforms should be stage informed. In advanced OSF, the submucosa is densely fibrotic and perfusion is reduced; dosing based on an “average patient” paradigm may therefore fail to achieve adequate local exposure at the lesion (Tekade et al., 2017; Dupare and Dhole, 2018; Tanaka et al., 2023). Stage stratified optimization of delivery strategies is needed to prevent under delivery in a remodeled tissue niche and to avoid insensitive readouts that obscure true therapeutic effects (Tanaka et al., 2023; Gondivkar et al., 2025; Murgod et al., 2014; Pandiar and Shameena, 2014).

### 1.3 Microenvironment driven self perpetuating fibrosis

A self reinforcing fibrotic niche underlies the progressive course of oral submucous fibrosis, resembling a maladaptive wound healing program sustained by aberrant biochemical cues and tissue mechanics (Tang et al., 2025a; Sharma et al., 2024a; Xu et al., 2023; Chang et al., 2025; Mayorca-Guiliani et al., 2025). Driven by this microenvironmental disequilibrium, persistent fibroblast activation and excessive extracellular matrix deposition become the central engines of fibrosis in OSF. Areca nut derived constituents, particularly the major alkaloid arecoline, can increase reactive oxygen species and promote activation of latent transforming growth factor-β1, thereby amplifying downstream profibrotic signaling (Tang et al., 2025a; Desai et al., 2025; Li M. et al., 2024; Sharma et al., 2024b; Liao et al., 2024; Hsieh et al., 2018; Kazemi et al., 2025). *In vitro*, arecoline exposed oral mucosal fibroblasts show marked activation of the TGF-β1 Smad axis with upregulation of profibrotic mediators such as CCN2 and EGR1, whereas epigallocatechin-3-gallate attenuates arecoline induced TGF beta 1 activation and limits ROS accumulation (Xu et al., 2023; Hsieh et al., 2018; Yang X. et al., 2024; Zhang et al., 2021; Saso et al., 2022).

In parallel, the high copper content in areca nut has been implicated in elevating lysyl oxidase activity, facilitating collagen crosslinking (Tang et al., 2025a; Warnakulasuriya and Chen, 2022; Trivedy et al., 1997; Raja et al., 2007). Consistent with this mechanism, LOX is markedly upregulated in OSF biopsy specimens; enzymatic crosslinking compacts and stiffens the collagen matrix, reshaping cellular mechanosensing (Khan et al., 2012; Mohapatra et al., 2023). Matrix stiffening then feeds back to further stimulate fibroblasts to secrete additional matrix components, creating a positive feedback loop. Hypoxia is another recurrent feature of OSF lesions, and increased HIF-1α expression has been reported in OSF tissues and during progression toward malignant transformation (R et al., 2024; Tilakaratne et al., 2008). Hypoxia can act synergistically with matrix stiffening to induce additional profibrotic and proangiogenic factors, further reinforcing fibrogenesis. Low grade chronic inflammation also contributes to microenvironmental maintenance, as leukocyte infiltration and elevated proinflammatory cytokines are commonly observed and can promote fibroblast proliferation and collagen synthesis through pathways such as NF-κB. Collectively, the OSF microenvironment constitutes an interconnected network in which soluble profibrotic signals, mechanical hardening, hypoxia, and inflammation mutually potentiate one another (Xu et al., 2023; Wang and Tang, 2022). Without effective intervention, this dysregulated repair state acquires pathological inertia, driving continued fibrotic accumulation (Zhao et al., 2022; Mascharak et al., 2024). Accordingly, targeting a single pathway is unlikely to dismantle the full vicious cycle, supporting the rationale for combinatorial approaches or smart, context responsive strategies designed to interrupt the self sustaining circuitry of fibrosis (He et al., 2024; Qin et al., 2023; Hao et al., 2022).

### 1.4 Limitations of current care and the unmet need for disease modification

With mechanistic insights and staging frameworks converging on tissue remodeling as the core pathological outcome, the therapeutic landscape of OSF can be reappraised through the lens of fibrosis control rather than symptom relief (Guideline for the Diagnosis, 2023). In routine practice, commonly used interventions primarily aim to palliate symptoms and dampen local inflammation, including intralesional corticosteroids alone or combined with systemic administration, hyaluronidase injections intended to soften fibrotic bands, and oral antioxidants to improve mucosal condition (Rai et al., 2023).

Despite their widespread use, these measures offer limited capacity to reverse established fibrosis and lack high quality evidence demonstrating a meaningful effect on long term disease trajectory (Guo et al., 2020). A systematic review has noted that while some agents may improve mouth opening and burning pain, overall effectiveness remains uncertain and further well designed randomized controlled trials are needed to clarify whether any intervention can reliably induce fibrosis regression (Shao et al., 2024; Kerr et al., 2011). The clinical imperative is sharpened by the malignant transformation potential of OSF, which some studies have reported to reach approximately 5%, underscoring the need for a strategy that is both safe for long term use and capable of slowing progression and ideally reducing malignant risk (Fedorowicz et al., 2008; Kujan et al., 2021). Symptom directed care is not equivalent to preventing fibrotic worsening or interrupting carcinogenic evolution (Dammling et al., 2022; Lorini et al., 2021). Consequently, patients continue to experience substantial impairment in quality of life, as restricted mouth opening compromises eating and social participation and imposes a sustained psychological burden (Manshi et al., 2023; Saalim et al., 2022). These limitations argue for a reconfiguration of efficacy assessment toward quantified functional and tissue endpoints (Gondivkar et al., 2025; Yang Y. et al., 2025; Arakeri and Brennan, 2018). Alongside functional measures such as maximal interincisal opening and validated pain or burning scores, tissue level readouts should capture changes in fibrotic band thickness and matrix composition through biopsy based assessment or imaging supported evaluation. Aligning such endpoints with delivery strategy design is essential to establish a target framework that is coherent from formulation engineering to clinical benefit. Taken together, OSF management is moving from supportive symptom control toward disease modification, a transition that will likely require advanced delivery approaches to maximize effective drug action within fibrotic tissue and innovative models and biomarkers to substantiate durable, long term gains.

## 2 Translational evidence supporting the use of OSF organoid models for mechanistic dissection and therapeutic efficacy prediction

### 2.1 Rationale for organoid models in OSF research

Because clinical outcomes in OSF do not always track proportionally with the extent of tissue remodeling, conventional two-dimensional cell culture systems and many animal models remain constrained in their ability to reproduce the human oral mucosal barrier, stromal composition, and disease tempo (Motgi et al., 2021; Bierbaumer et al., 2018; Tang et al., 2025b). This gap helps explain why candidate interventions that appear effective in preclinical settings may yield variable or equivocal results when translated to patients. Against this backdrop, advances in three-dimensional organoid technology offer a practical intermediate model that more closely approximates human tissue architecture (Loewa et al., 2023; Corrò et al., 2020; Zhou et al., 2021). Organoids are self-organized multicellular structures derived from adult stem cells or pluripotent stem cells that can recapitulate key genetic and histologic features of the tissue of origin *in vitro*. When combined with stromal cell co-culture, organoids can be expanded into a compact tissue ecosystem, enabling the interrogation of intercellular crosstalk and microenvironment dependent regulation (Fatehullah et al., 2016).

For OSF, the distinctive value of this platform lies in its capacity to model the interplay between oral epithelium and a fibrotic submucosal niche, while enabling quantitative assessment of drug accessibility and tissue level responses within a structured setting (Xu et al., 2023; Yang X. et al., 2024; Xu et al., 2024). For example, patient-derived oral epithelial organoids embedded in a collagen like matrix and exposed to profibrotic stimuli can exhibit coordinated changes in epithelial behavior and matrix deposition; subsequent introduction of a candidate therapy allows direct testing of whether these changes are prevented and through which mechanisms (Driehuis et al., 2020a). By coupling mechanistic validation with efficacy prediction in the same experimental system, organoid based approaches can improve human relevance and mitigate species related uncertainty inherent to animal models (Yang X. et al., 2024; Vlachogiannis et al., 2018; Wu et al., 2024). Recent literature has increasingly highlighted the role of organoid models in oral precancerous conditions and fibrotic disease contexts. Notably, a systematic review of OSF research models highlighted that limitations of existing preclinical models constrain robust evaluation of candidate therapies; in parallel, systematic reviews of oral organoids have advocated the establishment of patient-derived organoid biobanks to enable drug-responsiveness (drug sensitivity) testing (Zhang et al., 2025). Taken together, OSF research has a clear need for innovative platforms such as organoids to bridge the gap between traditional models and clinical populations, thereby strengthening translational evidence for the design and optimization of delivery strategies (Yang H. et al., 2024).

### 2.2 Oral organoid platforms and key considerations for model construction

At the level of model selection, epithelial organoids derived from patient oral mucosa have been successfully established for long term culture and functional interrogation, indicating that stable, expandable epithelial structures can be obtained directly from clinical tissue (Driehuis et al., 2020b). Driehuis et al. reported a method to culture organoids from normal human oral mucosa that enables sustained propagation without cellular immortalization, while preserving the stratified architecture and differentiation features of oral epithelium (Driehuis et al., 2020b; Driehuis et al., 2019). When deployed for pharmacologic response assessment, such patient-derived organoids can be used to quantify drug induced epithelial injury and the protective effects of candidate interventions (Millen et al., 2023). As noted above, an oral organoid model has been used to demonstrate that methotrexate induces substantial epithelial cell death, whereas pretreatment with a folate rescue agent reduces this damage in a dose dependent manner. This provides a measurable *in vitro* basis for evaluating biological effectiveness in local delivery concepts, because readouts such as organoid viability, inflammatory mediator release, and structural integrity can be directly quantified to judge formulation level protection (Takahashi et al., 2023; Hempt et al., 2021; Steinway et al., 2020; Tong et al., 2023).

From a construction standpoint, generating robust oral organoids typically requires alignment across several foundational elements (Seubert et al., 2021). The first is cell sourcing, where epithelial stem cells obtained from both lesional tissue and adjacent clinically normal mucosa are preferable to enable within patient comparisons and to resolve lesion specific drug effects (Driehuis et al., 2019; Wils et al., 2024). Organoid cultures are typically embedded in basement membrane extract hydrogels such as Matrigel, which provide a 3D scaffold with tissue-relevant biochemical ligands and biophysical properties that collectively support organoid survival, morphogenesis and differentiation (Kleinman and Martin, 2005; Sato et al., 2009). The third is the growth factor cocktail, which must be tuned to maintain proliferative capacity while permitting regulated differentiation (Yang L. et al., 2025). After establishment, organoids should be validated using morphology and lineage associated molecular markers to confirm appropriate differentiation and fidelity to the source tissue, thereby ensuring authenticity and stability of the model (Kim et al., 2020). Because inter batch and inter donor variability is common, experimental designs typically incorporate organoids from multiple donors, with standardized passaging and harmonized analytical pipelines to reduce technical variance (Carista et al., 2025; Dotti et al., 2022; Gehling et al., 2022; Velasco et al., 2019). Collectively, these practices enable the development of quality controlled, reproducible oral organoid systems that can serve as a reliable platform for subsequent evaluation of delivery strategies (Ahn et al., 2024).

### 2.3 Stepwise model complexification from epithelial organoids to a fibrotic niche

Once stable epithelial organoids are established, modeling fibrotic disorders such as OSF requires the deliberate incorporation of stromal cells, immune components, and matrix mechanics to more faithfully recapitulate the submucosal fibrotic niche (Papp et al., 2024). The prevailing approach is stepwise escalation of biological complexity toward assembled organoid systems that integrate multiple compartments in a controlled manner (Onesto et al., 2024). In practice, investigators have begun to co-culture fibroblasts with oral epithelial organoids, where the two components can self organize into rudimentary spatial architectures resembling epithelial stromal composites (Yang X. et al., 2024; Li X. et al., 2024). This configuration enables direct epithelial mesenchymal interaction and supports reconstruction of bidirectional signaling *in vitro* (Wu et al., 2024). As highlighted in the review by Wu et al., introducing fibroblasts together with immune cells into organoid platforms expands their utility for interrogating cellular crosstalk and microenvironment dependent regulation (Wu et al., 2024).

Culture strategies that preserve tissue like organization and salient niche features further advance model fidelity (Ootani et al., 2009). Air-liquid-interface conditions, for example, can promote deeper differentiation and yield more physiologic stratification (Shacham-Silverberg and Wells, 2020). In oral mucosal systems, air-liquid-interface culture has been associated with enhanced keratinization and strengthened barrier properties, offering a tractable means to approximate OSF relevant epithelial phenotypes such as thinning and hyperkeratosis (Kazemi et al., 2025; Lin et al., 2020; Dongari-Bagtzoglou and Kashleva, 2006). Methodological advances from tumor immunology also offer transferable blueprints for immune organoid integration (Tsai et al., 2018). For instance, approaches that add patient-derived peripheral blood T cells to tumor organoids have been used to evaluate immunotherapeutic responses (Votanopoulos et al., 2020). By extension, future OSF models could incorporate macrophages or T cells to examine how inflammatory immunity shapes fibrogenic programs and to quantify immune mediated modulation within a structured tissue context (Olawade et al., 2025; Wynn and Barron, 2010; Liu et al., 2022). Overall, progression from epithelial only organoids to systems that include stromal, immune, and mechanical determinants represents a disciplined trajectory of model complexification that brings experimental platforms closer to OSF pathology (Liu et al., 2025). The evolving complexity of OSF-relevant organoid models, along with their biological features and experimental applications, is summarized in Table 1. Notably, immune-enhanced configurations remain methodologically challenging in patient-matched settings: peripheral blood immune cells may not fully represent mucosal tissue-resident programs, and standard organoid expansion conditions can be incompatible with immune viability and function. Moreover, immune infiltration and cell–cell contact are constrained by 3D matrix and epithelial barrier properties, while donor-to-donor heterogeneity can introduce batch effects, underscoring the need for minimal reporting/QC items when interpreting such readouts. This evolution is directly relevant to delivery strategy evaluation, because it enables microenvironment informed screening that cannot be achieved in two-dimensional culture (Spagnol et al., 2023). Once fibroblast containing OSF organoids are available, nanoscale formulations can be tested for their ability to traverse fibrotic matrix and engage deep stromal targets; with added immune compartments, materials can also be evaluated for their impact on inflammatory responses (Yang X. et al., 2024; Li D. et al., 2024; Polak et al., 2024; Cahn et al., 2024; Huang et al., 2021). Such integrated readouts provide actionable guidance for optimizing delivery systems under conditions that better reflect the constraints and feedback loops of the fibrotic niche (He et al., 2023).

**TABLE 1:** Organoid models for OSF: composition, complexity, and use.

Organoid model	Cellular composition	Model complexity	Key biological features captured	Applications in OSF research
**Epithelial-only organoids**	Epithelial cells derived from oral mucosa; grown as self-organizing 3D epithelial spheroids	Moderate-3D multi-layered epithelial structure, but lacks supporting fibroblasts, vasculature, or immune cells	Mimics stratified oral epithelium architecture and differentiation; retains genetic and phenotypic traits of the source epithelium. Does not recapitulate stromal interactions	High-throughput drug or toxicity screening on patient-derived epithelium. Biomarker discovery for epithelial changes in premalignant lesions in isolation
**Fibroblast-attached organoids (FAO)**	Oral epithelial organoid clustered with primary oral fibroblasts co-embedded in 3D matrix (Matrigel) allowing direct cell–cell contact	High-bilayer co-culture More complex microtissue with stromal compartment, though still lacks blood vessels and immune cells	Reconstitutes epithelium-mesenchyme crosstalk: Epithelial signals activate fibroblasts, inducing myofibroblast phenotype and ECM production. Fibroblasts deposit collagen and express fibrosis-associated proteins as seen in OSF stroma. Replicates fibroplasia and epithelial atrophy seen in OSF, except vascular and immune components	Pathogenesis studies: Model OSF fibrogenesis *in vitro*, enabling dissection of signaling pathways between epithelium and fibroblasts Target discovery: Revealed stromal targets (THBS1) that mediate fibrosis and angiogenic suppression Drug testing: Evaluate antifibrotic compounds on a pseudo-tissue (readouts: collagen deposition, myofibroblast markers) Biomarker research: Study fibroblast-related biomarkers of disease progression in a controlled 3D context
**Vascularized FAO (vFAO)**	Oral epithelial cells + oral fibroblasts + endothelial cells co-cultured in 3D	Very high-multi-lineage assembled organoid. Contains epithelial layer, stromal fibroblasts, and forming microvessels	Simulates the fibrotic microenvironment with vasculature: Endothelial cells form sprouting vessel-like structures within the organoid. Models the aberrant angiogenesis of OSF Fibroblast-derived factors modulate endothelial growth overexpression of THBS1 suppresses microvessel sprouting in vFAO. Captures interactions among epithelium, stroma, and nascent blood vessels	Angiogenesis in fibrosis: Investigate how fibrosis impairs vascularization in OSF Therapeutic testing: Assess pro-angiogenic or antifibrotic therapies in a more physiologic 3D model Translational research: vFAO findings have informed *in vivo* approaches
**Immune-enhanced organoids**	No OSF-specific model reported yet very high—4-component co-culture	Sustain all cell types requires immune-compatible medium and time-windowed assays models chronic inflammatory milieu and immune–fibroblast/epithelial crosstalk	PBMC≠tissue-resident, media mismatch, limited 3D infiltration/contact, donor variability/batch effects (±alloreactivity if non-autologous) inflammation-fibrosis mechanism studies; screen immunomodulators (cytokines, collagen, myofibroblast markers)	Explore immune biomarkers linked to OSF progression

### 2.4 Translational phenotypic readouts and evaluation metrics

As organoid models become more complex, the phenotyping framework must expand in parallel to enable a rigorous assessment of delivery performance and biological effect (Wardwell-Swanson et al., 2020). Early reliance on simple viability measures is no longer sufficient; instead, integrated readout panels should capture structural integrity and functional state concurrently (Bozal et al., 2024). In epithelial fibroblast co-culture organoids, for example, outcomes can be quantified across epithelial layer continuity, epithelial differentiation status, stromal collagen accumulation, and fibroblast phenotypic shifts (Al Yazeedi et al., 2025). Together, these measures provide a composite view of fibrotic activity and epithelial health that more closely reflects the multidimensional pathology of OSF. Readouts that map directly onto fibrogenic activity in OSF can be organized around collagen crosslinking, hypoxia signaling, and mechanotransduction linked growth factor pathways (Xu et al., 2023; Chaudhary et al., 2015; Shetty et al., 2021; Singh et al., 2021). Such a design allows differences in intratissue drug exposure produced by competing delivery systems to be translated into interpretable biological response profiles (Giordano et al., 2016; Betge et al., 2022; Prasad et al., 2020; Mehta et al., 2012). If a nanoparticle formulation delivers EGCG with improved penetration in the organoid, a concordant reduction in collagen deposition and alpha smooth muscle actin signals would be expected; conversely, a system that remains confined to superficial layers may preferentially reduce epithelial injury while exerting limited influence on deeper stromal fibrosis (Niora et al., 2020). This exposure to response alignment is essential for determining whether a delivery improvement yields a *bona fide* antifibrotic effect rather than a partial, surface restricted benefit (Karolak et al., 2019). To align with the standardization agenda, we use the term readout panel to denote a predefined minimal metric set with prospectively specified decision cutoffs; candidates advance only if collagen deposition is reduced by at least 30% and alpha smooth muscle actin signal is reduced by at least 25% relative to the fibrotic control, while maintaining epithelial viability above 80%.

When organoids are used for formulation screening or efficacy prediction, standardization becomes a methodological requirement rather than an optional refinement (Puschhof et al., 2021). Expert consensus has emphasized the need to define response thresholds, reporting conventions, and other parameters in drug sensitivity testing to enable cross study comparability (Hafner et al., 2017). For OSF delivery research, this translates into a shared organoid evaluation protocol in which nanomedicine candidates are assessed using harmonized fibrosis oriented endpoints and predefined decision criteria, thereby accelerating evidence aggregation and facilitating convergence toward actionable consensus (Wensink et al., 2021; Rønnow et al., 2020; LeSavage et al., 2022).

### 2.5 Standardization and reproducibility agenda

Standardization and reproducibility are prerequisites for positioning organoids as credible evidence generators within a delivery focused translational pipeline (Moon et al., 2024). Variability can arise from multiple sources intrinsic to organoid workflows, including heterogeneity in donor tissue baseline states, batch to batch differences in matrix materials, and subtle fluctuations in culture conditions, any of which may materially shift experimental outcomes (De Witte et al., 2020; Glass et al., 2024; Guo et al., 2024). To mitigate these risks, unified norms are needed at the levels of methodological reporting and quality control (Pamies et al., 2024). In published organoid studies, detailed disclosure of donor characteristics, medium composition, and passage history enables independent replication and more transparent appraisal of evidentiary strength (Mohammadi et al., 2021). Moving toward engineering scale up and clinical facing applications, routine incorporation of defined media formulations, standardized passaging strategies, genetic stability surveillance, and phenotypic drift monitoring becomes essential to ensure that organoid platforms evolve from laboratory proof of concept into evidence production systems that are compatible with regulatory and industrial expectations (Renner et al., 2015; Jensen and Little, 2023). Operational standardization has already been demonstrated as feasible in adjacent fields (Abdellatif et al., 2025). A large collaborative effort in cancer organoids, for example, established standard operating procedures spanning biobank construction, drug sensitivity testing, and data sharing, thereby providing a template for how multi center alignment can be implemented in practice (Palechor-Ceron et al., 2019; Francies et al., 2019). Analogously, the OSF community would benefit from a coordinated network that harmonizes protocols and reporting to reduce between laboratory inconsistency and enable cumulative evidence building (Sandoval et al., 2024). Near clinical deployment, an expert consensus published in 2024 specifically addressed the normalization of organoid based drug sensitivity testing and offered guidance on defining interpretable workflows and output formats (Sandoval et al., 2024). That consensus also underscored preclinical validation requirements, including control of systematic biases and resolution of key translation gaps such as inter laboratory concordance and the correlation between *in vitro* readouts and patient level responses (Vlachogiannis et al., 2018; Chen Y. et al., 2025; Xiang et al., 2024). Only by consolidating this standardization agenda can organoids be applied efficiently to screen and optimize delivery strategies, while ensuring that resulting conclusions withstand independent verification and regulatory scrutiny (Ahn et al., 2024; Xiang et al., 2024).

## 3 Design framework and implementation pathway for EGCG delivery systems in OSF

### 3.1 Antifibrotic basis of EGCG and key delivery barriers

Within the complex fibrotic circuitry of oral submucous fibrosis (OSF), epigallocatechin-3-gallate (EGCG), a major green tea polyphenol, has emerged as a multitarget antifibrotic candidate. Mechanistically, EGCG has been shown to suppress TGF-β-mediated transcriptional responses and to downregulate multiple downstream profibrotic effectors implicated in fibroblast activation (Hsieh et al., 2017). By activating the Nrf2 antioxidant program, EGCG can mitigate TGF-β1-induced epithelial-to-mesenchymal transition, with antifibrotic effects documented in renal fibrosis models (Wang et al., 2015a; Wang et al., 2015b). Consistent with its anti-inflammatory and antioxidative profile, EGCG markedly reduces the production of interleukins and other proinflammatory mediators, as well as type I collagen, in studies using fibroblasts derived from nasal polyps (Kang et al., 2014; Lin et al., 2008; Kim et al., 2006). Collectively, these pleiotropic actions map onto several central pathogenic nodes in OSF, including oxidative stress, LOX activity, and TGF-β signaling (Wei et al., 2021; Guo et al., 2025). In an arecoline-related OSF *in vitro* setting, Hsieh and colleagues further reported that EGCG dose-dependently attenuated arecoline-induced reactive oxygen species accumulation and latent TGF-β1 activation, providing mechanistic plausibility for EGCG as a disease-modifying agent in OSF (Hsieh et al., 2018).

However, the physicochemical liabilities of EGCG create substantial barriers to clinical translation. EGCG is prone to rapid auto-oxidation and degradation under neutral to alkaline conditions, resulting in poor intrinsic stability. In parallel, its oral bioavailability is notably low, and peak systemic concentrations after ingestion are often less than one-tenth of the *in vitro* effective range (Fangueiro et al., 2014). These dual constraints, chemical instability and limited *in vivo* exposure, have been repeatedly highlighted, fueling concerns about the reproducibility of EGCG efficacy across studies (Cai et al., 2018). Put differently, whether EGCG can deliver meaningful benefit in OSF largely depends on enabling a sufficient number of active EGCG molecules to reach the diseased mucosa and to persist there for an adequate duration, thereby making delivery strategy a prerequisite rather than an adjunct (Mehta et al., 2025; Mehta et al., 2024). From an *in vivo* perspective, early animal data provide encouraging signals: in an arecoline-induced rat model of OSF, EGCG administered at an appropriate dose and via a suitable route reduced collagen deposition, improved mouth opening, and lowered TGF-β1 levels (Gao et al., 2025; Mehta et al., 2023). Yet these findings also underscore the evidentiary gap that currently limits translation, namely, the lack of systematic data linking administration route, tissue exposure, and pharmacodynamic response (Lai et al., 2009). Accordingly, before advancing EGCG toward clinical application in OSF, delivery remains the principal bottleneck to resolve; only with a credible solution to this constraint can EGCG’s multitarget antifibrotic potential be converted into reproducible clinical efficacy.

### 3.2 Design principles and evaluation criteria for delivery systems

With EGCG’s pharmacological promise and exposure barriers clearly defined, OSF focused delivery can be framed around three interdependent objectives: prolonging residence and promoting mucosal penetration at the lesion site to achieve an effective intratissue concentration, while tuning release kinetics to sustain local activity over time. Because the oral mucosa presents a distinctive barrier architecture and is continuously wetted and rinsed by saliva, topically applied formulations are readily diluted, swallowed, and lost, making enhanced mucoadhesion and wash resistance a logical starting point in system design (Shaikh et al., 2011; Chinna Reddy et al., 2011). Accordingly, many nanocarriers and local dosage forms incorporate adhesion promoting strategies, such as leveraging cationic polymers to electrostatically interact with negatively charged mucins, or adopting a dry film format that conforms to and remains apposed to the mucosal surface after hydration (Ways et al., 2018). Yet residence alone is insufficient, as the payload must also traverse the epithelium to reach the submucosal fibrotic compartment where activated fibroblasts reside; delivery platforms therefore need to explicitly manage the trade off between adhesion and permeation (Vasquez-Martínez et al., 2023). Excessive surface adhesion can sequester EGCG within superficial layers and limit exposure to deeper stromal targets, whereas designs that prioritize penetration alone may be cleared rapidly during transit or diffuse away, ultimately shortening local residence and diminishing site specific exposure (Mehta et al., 2025; Schneider et al., 2017).

Evaluating the effectiveness of a nanocarrier or biomaterial delivery platform must be multidimensional rather than reduced to a single metric such as an *in vitro* release profile. A practical starting point is to quantify mucoadhesive strength and *ex vivo* residence time, namely, how long the formulation remains on mucosal tissue before being removed under simulated salivary flow (Chinna Reddy et al., 2011; Madsen et al., 2013). The next step is to characterize penetration routes and depth, for example, by mapping the spatial distribution of drug-loaded nanoparticles on tissue sections, and by comparing performance with versus without permeation enhancers (Sohi et al., 2010; Ostrowski et al., 2015). In parallel, intratissue drug exposure should be measured dynamically by profiling time dependent EGCG concentrations in target tissue, such as through tissue sampling in animal models, to verify that the system genuinely increases local bioavailability rather than merely altering release kinetics (Lin et al., 2007; Marchand et al., 2016; Zhang et al., 2019). Ultimately, these exposure and distribution parameters should be linked to biological responses, including whether higher tissue concentrations translate into more pronounced reductions in collagen deposition (Jenkins et al., 2017). Only when formulation metrics are explicitly aligned with antifibrotic efficacy readouts can an optimal delivery strategy be selected. Notably, recent reviews of EGCG delivery have consolidated several approaches with reproducible benefits, including encapsulating EGCG in lipidic or polymeric carriers to improve stability and employing surface functionalization to enable mucosal localization and site biased release (Granja et al., 2017; Farabegoli and Pinheiro, 2022). These strategies, validated across other disease contexts, provide a useful starting set of design hypotheses for OSF (Hu et al., 2021). However, OSF specific translation still requires deliberate bridging between formulation parameters and fibrotic endpoints, as emphasized above: multidimensional evaluation in intermediate models is needed to convert “improved stability” into evidence of “reversal of fibrotic signaling”.

### 3.3 Strategy spectrum of nanodelivery platforms

With enhanced local exposure as a shared objective, a spectrum of nanodelivery platforms can be tailored for EGCG, each offering distinct functional trade-offs (Sun et al., 2024). Driven by materials chemistry and interfacial design, polymeric nanoparticles typically provide robust encapsulation and tunable release, often enabling high EGCG entrapment efficiency (Yang et al., 2022). Lipid-based nanosystems, by virtue of favorable membrane compatibility and small size (commonly <300 nm), can facilitate epithelial permeation (Gugleva and Andonova, 2023). Hybrid architectures further extend this design space, for example, by combining a mucoadhesive polymer core with a mucus-penetrating outer shell to reconcile mucosal retention with tissue penetration (Subramanian et al., 2022). These approaches diverge across key performance dimensions, including drug loading and encapsulation efficiency, release kinetics, and the mode and strength of interactions with the oral mucosa (Jhaveri et al., 2021). A comparative overview of these delivery platforms in terms of mucoadhesion, penetration, EGCG stability, and antifibrotic efficacy is provided in Table 2. For comparability, we standardized the key parameters reported in Table 2, including particle size, dispersity index, zeta potential, encapsulation efficiency or drug loading, key release profile features, stability, evidence for mucoadhesion or tissue penetration, and *in vivo* or organoid based functional readouts, with NR used when not reported.

**TABLE 2:** Nanoformulation strategies for EGCG delivery in oral submucous fibrosis (OSF). Comparison of carrier types and key performance parameters (Standardized fields include particle size, dispersity index, zeta potential, encapsulation efficiency or drug loading, release profile features, stability, mucoadhesion or tissue penetration evidence, and *in vivo* or organoid functional readouts).

Formulation (carrier type)	Mucoadhesion	Penetration depth	EGCG stability	Antifibrotic efficacy
**Conventional EGCG**	Low-rapidly cleared by saliva, short mucosal contact	Low-limited mucosal permeation	Poor-EGCG prone to oxidation and degradation at physiological pH	Moderate – native EGCG can suppress fibrosis markers *in vitro*/*in vivo*, but brief exposure limits its therapeutic impact
**Polymeric NanoCubogel**	High-strong mucoadhesion via hydrogel matrix and positive surface charge; formulation remains at lesion for extended duration	Improved- nanoscale size and +46 mV surface facilitate penetration into epithelium and submucosa	High-encapsulation protects EGCG from degradation, enabling sustained release	High-significantly reduces TGF-β_1_ and collagen deposition in OSF models, restoring nearly normal mucosal histology; no local toxicity observed
**NanoCuboSpray**	High-forms a viscous, adhesive film on buccal mucosa, ensuring prolonged contact	High-intimate film contact plus nanoparticle permeation yield deep mucosal penetration and retention	High-EGCG encapsulated in film matrix is shielded from degradation	Very high-achieved superior antifibrotic outcomes in rats, outperforming standard OSF therapy while showing a favorable safety profile

In oral mucosal delivery, an explicit trade-off emerges between mucus penetration and mucosal adhesion. One nanomedicine review notes that decorating nanoparticle surfaces with ieties can reduce viscoadhesive interactions with salivary mucins and thereby improve transport across the mucus layer, albeit at the cost of diminished residence on the mucosal surface (Lai et al., 2007; Li et al., 2026). By contrast, positively charged surfaces or adhesive ligands tend to immobilize particles rapidly at the entry region, limiting deeper access to the underlying tissue (García-Díaz et al., 2018). A layered delivery concept has therefore been proposed, whereby an adhesive matrix first anchors the nanomedicine at the target site, and—upon patch dissolution—releases penetration-competent nanoparticles to migrate into submucosal compartments (Giovino et al., 2013). From a pragmatic translational perspective, prior successes in oral and sublingual nanodelivery provide actionable precedents for OSF formulation choices (Franz-Montan et al., 2007; Bahraminejad and Almoazen, 2025). A review of transmucosal nanotherapeutics summarizes physiological determinants of absorption and local efficacy, including oral pH, salivary flow rate, and the degree of epithelial keratinization, all of which are particularly pertinent in OSF (Abdul Khader and Dyasanoor, 2015; Patel et al., 2011). By leveraging design principles established for sublingual lozenges and buccal patches, EGCG platforms can be more rationally optimized to match the distinctive oral microenvironment of OSF patients.

### 3.4 Innovations in local biomaterials and dosage forms

As the delivery objective shifts from improving solubility to maximizing tissue residence and achieving controllable local exposure, innovations in biomaterials for intraoral administration become particularly consequential. Relative to mouthrinses or sprays that are readily cleared, dosage forms such as gels, patches, and films can leverage mucoadhesion and *in situ* film formation to substantially mitigate salivary washout. A mucoadhesive oral gel, for instance, incorporates viscoelastic polymers that spread over the lesion and set into a thin gel layer, thereby sustaining drug release while buffering dilution by Špiljak et al. (2025). By contrast, a mucoadhesive patch is typically fabricated from slowly dissolving polymers; once applied to the buccal or sublingual mucosa, it maintains intimate contact with the epithelium and promotes transepithelial drug transport into the underlying tissue (Jacob et al., 2021).

Localized biomaterial-based delivery is especially attractive for oral submucous fibrosis (OSF), because the disease is anatomically confined to the oral cavity and maintaining a high drug concentration at the lesion can enhance efficacy while limiting systemic exposure (Cheng et al., 2023). Notably, OSF-focused studies have begun to evaluate topical EGCG formulations against both pathological and functional endpoints (Mehta et al., 2025). In a study by Acharya et al., EGCG was formulated into a bioadhesive hydrogel and applied daily to fibrotic lesions in a rat model of oral fibrosis; compared with a conventional treatment, the EGCG hydrogel produced greater improvement in mouth opening and reduced tissue TGF-β1 expression and collagen burden (Mehta et al., 2023). A more recent advance extended this concept by developing a “NanoCuboSpray,” in which EGCG nanoparticles are incorporated into a film-forming spray (Mehta et al., 2025). In a rat OSF model, this spray generated a compact drug film on the mucosa and provided sustained EGCG release, yielding therapeutic effects superior to EGCG gel alone and comparable to, or even exceeding, those observed with intralesional steroid injection. These studies offer an important evidentiary entry point by explicitly linking engineering parameters to disease-relevant outcomes: adhesive testing can substantiate prolonged film residence, permeability experiments can quantify penetration depth of nanoformulated EGCG, and both can be aligned with measurable improvements in fibrotic remodeling (Pinto et al., 2020; Smart, 2005). For translational writing, the rationale for material selection should therefore be anchored to quantifiable, reproducible metrics rather than generic efficacy claims. In other words, instead of broadly stating that a material “enhances EGCG efficacy,” the report should specify which metrics improve, such as a defined adhesive strength (Pa), a 24-h cumulative release fraction (X%), and a mucosal penetration depth (Y μm), and clarify how these parameters map onto OSF endpoints. Such specification reduces reliance on empirical formulation narratives and makes the mechanistic basis of precision delivery transparent to the reader.

### 3.5 Stimuli-responsive delivery strategies geared toward combination therapy

Given the highly dynamic oral milieu, stimuli-responsive materials are increasingly being explored to enable on-demand drug release and context-adaptive performance (Hua, 2019). In inflammation-associated disease settings, local elevations in peroxides and concomitant decreases in tissue pH constitute actionable microenvironmental cues; delivery vehicles capable of converting these stimuli into controlled release are well positioned to improve spatiotemporal precision. For example, a smart hydrogel developed for periodontitis undergoes accelerated degradation in hydrogen peroxide-rich conditions, rapidly liberating an antioxidant payload to meet the demands of acute inflammatory episodes, while maintaining sustained release in healthy tissue to reduce unnecessary drug depletion (Zhu et al., 2025; Huang et al., 2025). This design logic is directly extensible to oral submucous fibrosis (OSF): carriers engineered to sense the heightened oxidative stress or low-oxygen niche of active fibrotic lesions could intensify drug release within fibrogenic regions, thereby improving therapeutic selectivity (Guo et al., 2025). Notably, the periodontal literature has systematically summarized stimuli-responsive hydrogels for local delivery, covering feasible triggering modalities including temperature, light, pH, and reactive oxygen species (Wang et al., 2023). Together, these studies demonstrate that rational chemical modification can embed sensing elements into carrier matrices, programming condition-dependent physicochemical transitions that govern release behavior.

For OSF, these methodological paradigms are readily transferrable, including the development of redox responsive nanogels. In physiologic conditions, such systems can remain structurally stable; upon entry into ROS enriched fibrotic tissue, they disassemble rapidly to release EGCG, thereby amplifying local antioxidant and antifibrotic activity. A complementary, forward looking direction is combination oriented therapy. Although EGCG exerts pleiotropic effects, a single agent is unlikely to intercept every node of the OSF pathological network (Ye et al., 2017). Lessons from other fibrotic disorders indicate that co administration of two or more antifibrotic or anti inflammatory components can yield synergy (Wuyts et al., 2014). In rat liver fibrosis models, a combined regimen comprising taurine, EGCG, and genistein demonstrated stronger antifibrotic efficacy than EGCG monotherapy, with reported reductions in hepatic hydroxyproline content and collagen deposition and downregulation of profibrotic activation markers including α-SMA and the TGF-β1/Smad3 axis. Mechanistically, iTRAQ-based proteomics in activated rat hepatic stellate cells further implicated coordinated suppression of fibrosis-linked metabolic programs, highlighting decreased expression of the glycolytic enzyme hexokinase-2 (HK2), which supports the rationale that the combination may converge on both collagen production and aerobic glycolysis-related remodeling (Zhuo et al., 2012). This evidence base motivates a combination guided OSF delivery strategy, such as pairing EGCG with low dose first line corticosteroids to strengthen fibrosis reversal while mitigating steroid related adverse effects; however, the safety margin and incremental benefit in OSF require cautious validation (Alora Veedu et al., 2015). At present, there are no clinical data on EGCG based combination regimens for OSF, making it necessary to first interrogate compatibility, synergy indices, and potential toxicity ranges in organoid and animal models (Gao et al., 2025). Only when combination treatment demonstrates a material advantage without added risk in these systems would progression to clinical trials be justified (International ethical guidelines, 2016). Collectively, stimuli responsive materials and combination therapy constitute two major extensions of future OSF delivery strategies, with the former prioritizing spatiotemporal precision and the latter expanding mechanistic coverage (Ran et al., 2024). Integrated with the nano and biomaterial innovations outlined above, these approaches may further enhance the disease modifying potential of EGCG and related agents in OSF.

## 4 Organoid-enabled translational pathway for delivery systems and clinical implementation

### 4.1 Organoid-based formulation screening and optimization strategy

When multiple carrier and material routes coexist, organoid platforms provide an efficient screening logic: applying distinct delivery formulations to organoids derived from the same patient or cultured under identical conditions, and determining which option yields the most pronounced and comprehensive improvement in fibrotic signatures. This design converts formulation heterogeneity into directly comparable biological readouts. Specifically, if formulation A delivers EGCG nanoparticles with greater stability and superior penetration than formulation B, organoids should exhibit correspondingly lower collagen accumulation and reduced expression of fibroblast-activation markers in the A-treated group relative to the B-treated group. Conversely, if the two groups achieve similar outcomes, the marginal value of the proposed modification is likely limited. Head-to-head comparisons across delivery systems within the same tissue background help eliminate cosmetically appealing but biologically inconsequential designs, thereby prioritizing strategies that meaningfully enhance therapeutic performance. In practice, oral mucosal organoids have already been used to evaluate drug toxicity and protective interventions. For instance, organoid models of methotrexate-induced oral mucosal injury have successfully captured the protective effects of folate-based agents, underscoring that oral organoids can generate interpretable outputs along the epithelial injury-repair axis and can therefore serve as an experimental scaffold for OSF formulation screening. As screening goals move beyond epithelial protection toward modulation of the fibrotic microenvironment, epithelial organoids should be progressively integrated with stromal components to establish assembled organoids of the kind discussed in Section 2.3. Such systems enable simultaneous assessment of epithelial and matrix responses to a given delivery platform. For example, in fibroblast-containing organoids, screening EGCG nanoformulations should not only document whether epithelial cell death is reduced, but also quantify changes in collagen and α-SMA.

Formulation screening should also be coupled to analytic approaches that reflect intratissue drug distribution (Han et al., 2008). Practical options include tracking EGCG diffusion within organoids using fluorescent labeling, or quantifying introrganoid concentration gradients with microscale probes. These measurements ensure that optimization conclusions close the loop with delivery design: establishing not only that formulation A performs better, but also why it performs better, for example, higher intratissue exposure and longer retention. This mechanistic anchoring is essential for guiding subsequent formulation refinement and dose-setting. Overall, organoid-enabled screening connects engineering parameters to efficacy outcomes within a standardized biological platform, substantially improving both screening efficiency and translational relevance.

### 4.2 Biomarker development and responder stratification

OSF displays substantial inter-patient heterogeneity in exposure burden, disease stage, and coexisting oral pathology or malignant transformation risk. Accordingly, both clinical trial design and downstream implementation require robust stratification frameworks (Superchi et al., 2022). An exposure and stage-driven scheme can serve as an initial stratifier, allowing treatment effects to be evaluated separately in, for instance, heavily exposed late-stage OSF versus mildly exposed early-stage OSF. Pooling treatment-sensitive and treatment-insensitive patients can dilute the observed average treatment effect and reduce statistical power; enrichment/stratified analyses mitigate this dilution by evaluating effects within more homogeneous subgroups (Yin et al., 2017). Finer responder definition, however, may hinge on biomarker-guided enrichment. In principle, informative biomarkers should reflect the intensity of ongoing fibrotic activity and predict therapeutic responsiveness. Mechanistically, candidates can be prioritized along collagen cross-linking, hypoxia, and mechanotransduction axes: LOX activity and downstream cross-linking products index matrix stiffening; HIF-1α reflects local hypoxia and angiogenic remodeling; and YAP/TAZ subcellular localization reports cellular mechanostress (López et al., 2010). A practical workflow is to quantify these markers in paired patient biopsies and patient-derived organoids to test concordance, such that a biopsy with high LOX and HIF-1α expression is mirrored by the corresponding organoid phenotype (Cacciatore et al., 2023; Yin et al., 2026).

Next, it is necessary to determine whether biomarker-high cases exhibit a more pronounced response to EGCG delivery, for example, whether LOX-high organoids show greater sensitivity to collagen reduction when treated with an EGCG nanoformulation (Wensink et al., 2021). If such an association is confirmed, these biomarkers could be used prospectively to enrich for the patient subset most likely to benefit. A central advantage of organoids is that they enable a direct linkage between biomarker signals and drug response. Conventionally, biomarker studies infer relationships by correlating marker abundance in patient histology with clinical outcomes, without an intervening functional validation layer (Mandrekar and Sargent, 2009). By contrast, organoids allow head-to-head testing of the same delivery strategy in biomarker-high versus biomarker-low models under controlled conditions, converting cross-sectional correlation into functional evidence (Van Renterghem et al., 2023). For instance, if EGCG delivery substantially attenuates fibrotic readouts in an HIF-1α-high subgroup but yields only modest effects in HIF-1α-low organoids, one may reasonably infer that patients with a hypoxia-dominant lesion microenvironment represent a plausible responder subpopulation (Chaudhary et al., 2015). Analogous paradigms have been reported in other indications: one study used patient-derived organoids to predict drug sensitivity in colorectal cancer and, through retrospective analyses, identified gene-expression differences associated with therapeutic efficacy (Ooft et al., 2019). Therefore, in OSF, integrating organoid-based pharmacology with biomarker profiling offers a pragmatic route to accelerate biomarker validation and responder definition within a simulation-validation closed-loop paradigm, which is pivotal for future precision-treatment development (Ma et al., 2025).

### 4.3 Building a translational evidence chain and de-risking engineering bottlenecks

Before entering the clinic, an ideal delivery strategy should be underpinned by an auditable translational evidence chain that links engineering design decisions to clinical benefit (Kimmelman et al., 2024). This chain should span formulation physicochemical attributes, intratissue exposure and distribution, mechanistic readouts, and functional endpoints. Conceptually, it functions as a sequence of interdependent links, where failure of any single element can compromise the whole. A structured mapping of delivery design elements to their expected biological impact and patient level benefit, together with potential bottlenecks and validation strategies, is provided in Table 3. Organoids can serve as a human relevant filter that anchors the intermediate step of relevance validation and candidate triage in the preclinical pipeline. To improve practicality, we add a concise organoid guided decision workflow for candidate formulation selection. In brief, candidates are benchmarked under the same dosing schedule in matched donor derived organoid cohorts, and prioritized only when they simultaneously meet a minimal metric set spanning material quality, tissue exposure, mechanism relevant efficacy, and local safety, with manufacturability and deployability considered at the final selection step. The minimal metric set includes EGCG stability and release behavior, organoid level exposure metrics such as retention and penetration, mechanism relevant readouts such as collagen reduction and myofibroblast activation markers, and basic safety readouts including viability and epithelial integrity surrogates. Formulations are compared by direct side by side performance across multiple donors, and selection is based on consistent benefit without added toxicity, while key SOP elements for cross center reproducibility include harmonized biopsy handling timelines, defined organoid passage range and matrix lot tracking, standardized stimulation and dosing windows, and a shared readout and metadata reporting template. These mechanistic readouts were selected because they map onto the core pathological axes that define OSF persistence. Collagen abundance reflects the net ECM accumulation state, whereas α-SMA reports myofibroblast activation that directly drives matrix production and contractility. LOX represents a key “stabilization” node, as collagen crosslinking increases matrix stiffness and reduces reversibility, thereby locking fibrotic architecture into a mechanically reinforced state. Hypoxia-related markers capture the vascular-compromised, diffusion-limited microenvironment that emerges with progressive fibrosis and can further reinforce profibrotic transcriptional programs. Mechanotransduction-associated markers. Importantly, these endpoints are quantitative and scalable in organoid-based assays, enabling head-to-head formulation benchmarking: delivery parameter choices can be optimized against a unified pharmacodynamic panel that reports whether a candidate formulation truly attenuates the fibrotic set-point rather than producing superficial epithelial-only effects (Chaurasia et al., 2019). Serving as a human-relevant filter, organoids can anchor the intermediate step of relevance validation and candidate triage in the preclinical pipeline (Ma et al., 2025). In practice, organoids enable screening of formulation libraries to verify lesion relevant exposure and mechanism aligned responses, so that only the best performing candidates advance to animal studies and clinical trials (Chen L. et al., 2025; Zuo et al., 2025). This strategy can substantially reduce the number of candidates entering clinical validation and thereby improve the probability of success (Jiang et al., 2025). In parallel with evidence generation, manufacturability and regulatory acceptability frequently determine whether a delivery concept can be implemented at scale. Key translational bottlenecks include material provenance and lot to lot consistency, quality control, stability and storage requirements, and safety assessment for long term local use in the oral cavity. Each of these constraints should be systematically resolved before clinical testing. For example, a mucoadhesive patch that requires cold-chain storage and remains viable for only a few days would be impractical for routine clinical deployment, necessitating reformulation, incorporation of appropriate preservatives, or substitution with more stable materials (Rodansky et al., 2015). Likewise, if industrial scale-up of a nanocarrier leads to broad particle-size distributions and reduced encapsulation efficiency, process development with formulation engineering teams becomes essential to restore critical quality attributes. Lessons can be drawn from the industrialization of established oral mucosal products, where some patches progressed from laboratory prototypes to commercial products through formulation simplification and excipient substitution to align with FDA-accepted safety frameworks such as GRAS practices.

**TABLE 3:** Translational “evidence chain” linking EGCG delivery design features to biological outcomes in OSF, with bottlenecks and validation methods.

Delivery design feature	Intended effect on delivery	Resulting biological outcome	Patient-level benefit	Key bottlenecks and validation
**Mucoadhesive formulation**	Prolonged residence at oral lesion-formulation adheres to mucosa, resisting wash-out by saliva	Increased local EGCG exposure in tissue leads to stronger suppression of fibrotic activity. Extended contact also improves drug penetration into subepithelial layers	Sustained drug action at the site translates to more effective symptom relief and less frequent dosing	**Bottleneck:** Adhesion can be reduced by saliva flow and tongue movement **Validation:** *Ex vivo* mucosal retention assays confirm prolonged adhesion; *in vivo*, a mucoadhesive EGCG spray showed enhanced therapeutic effect correlating with longer mucosal contact. Patient acceptability studies ensure the formulation remains in place without discomfort
**Nanoparticle encapsulation**	Protects EGCG from degradation and provides controlled release of the drug. Nanocarrier matrix shields EGCG from oxidation and enzymatic breakdown; gradual release maintains therapeutic levels	Higher sustained concentration of active EGCG in the lesion over time. Continuous presence of EGCG produces persistent antifibrotic effects. In models, nano-EGCG maintained lower fibrotic markers for longer vs. free EGCG.	More durable treatment effect with potentially fewer applications needed. Enhanced efficacy and improved safety were observed with nano-encapsulated EGCG	**Bottleneck:** Achieving high drug loading and proper release kinetics in the nanoparticle-insufficient loading or too rapid release could diminish benefits. Ensuring formulation stability during storage is also critical. **Validation:** *In vitro* stability tests and release profile studies guide optimization. Efficacy is confirmed *in vivo*: EGCG nanoparticles significantly outperformed unencapsulated EGCG in reducing TGF-β_1_ and collagen in an OSF rat model
**Cationic nanoscale particles**	Enhanced mucosal penetration and cellular uptake. Small, positively charged particles penetrate the oral epithelium and diffuse into the fibrotic submucosa more effectively. The cationic surface promotes interaction with the negatively charged mucosal membrane, improving transmucosal delivery	EGCG reaches deeper fibroblast-rich layers of the lesion and enters target cells. This yields a more pronounced reduction in collagen deposition at the lesion core. Greater intralesional drug distribution was correlated with stronger antifibrotic outcomes in preclinical studies	Potential to regress established fibrotic bands and improve oral opening more substantially than surface-limited treatments. Deep tissue penetration means even advanced OSF areas receive therapeutic EGCG, improving overall treatment response	**Bottleneck:** Dense collagenous ECM in OSF can still impede particle diffusion; overly high positive charge may cause mucin binding or local irritation. An optimal size/charge balance is needed to maximize penetration without toxicity **Validation:** *Ex vivo* penetration studies demonstrate depth of tissue infiltration. 3D organoid models and imaging confirm nanoparticle uptake by fibroblasts in the interior of the tissue model. Tuning surface charge significantly boosted permeation efficiency, validating the design approach
**Sustained-release system**	Continuous delivery of EGCG over an extended period instead of a rapid burst. Formulation releases the drug at a controlled rate, maintaining therapeutic levels and avoiding quick clearance	Prolonged inhibition of pro-fibrotic signaling in the tissue. By keeping TGF-β1 and other fibrosis drivers suppressed for longer durations, sustained-release EGCG allows the tissue’s remodeling processes to proceed. Prevents “rebound” fibrosis that could occur if drug levels drop off	Less frequent application and more consistent symptom improvement. A long-acting EGCG formulation could provide round-the-clock antifibrotic action, leading to continuous improvement in mouth opening and reduction in burning sensation over the dosing interval	**Bottleneck:** Difficulty in matching release rate to the optimal therapeutic window – too fast may require frequent re-dosing; too slow may underdose the tissue. Individual variability in lesion thickness and saliva conditions can affect drug release dynamics **Validation:** *In vitro* release kinetics assays are used to adjust polymer composition for desired release profiles. *In vivo*, monitoring of EGCG levels in oral tissues and corresponding fibrosis markers over time helps confirm that the release profile translates to sustained pharmacologic effect. Clinical pilot studies would validate improved outcomes with a sustained-release formulation versus immediate-release

At the methodological level, the expert consensus referenced above also articulates process-oriented recommendations for organoid-based drug sensitivity testing. In effect, these recommendations provide a template for the standardized translational pathway required for clinical adoption, namely, how to convert research-grade screening into reproducible clinical decision support. For example, the consensus advocates harmonized efficacy endpoints and prespecified decision criteria so that organoid sensitivity results generated across centers are directly comparable (Xiang et al., 2024). Applied to OSF delivery, this implies that if multiple groups ultimately use organoids to evaluate EGCG formulations, they should adopt standardized metrics such as the percentage reduction in collagen, and strengthen evidentiary credibility through third-party, blinded validation, thereby improving both the trustworthiness of the findings and their acceptability to regulators. Only a rigorous, auditable evidence chain can persuade clinicians and regulatory authorities that a given delivery strategy is truly effective and warrants deployment in patients.

### 4.4 Advantages, outlook, and conclusions

After integrating EGCG’s pharmacological promise with the practical constraints of delivery, its therapeutic outlook in oral submucous fibrosis becomes more tractable. Reproducible disease modification will depend less on nominal potency than on whether nanocarriers or local biomaterials can establish stable, sufficiently high intratissue exposure at the lesion (Jansson-Löfmark et al., 2020). Driven by sustained local availability, EGCG’s convergent actions on TGF-β signaling, oxidative stress, and inflammation could plausibly slow fibrotic progression and, in some patients, enable partial reversal; conversely, inadequate delivery will render even robust bioactivity clinically silent (Mehta et al., 2024). Encouragingly, several EGCG nanoformulations have produced favorable signals in animal models, supporting cautious optimism for clinical translation (Cano et al., 2019). Yet clinical OSF presents a more heterogeneous and perturbed mucosal landscape, where ulceration, secondary infection, and other microenvironmental disruptions may materially reshape retention, penetration, and release kinetics, thereby altering performance in ways preclinical systems do not fully capture (Rao et al., 2020). Accordingly, these delivery platforms require rigorous clinical evaluation of both efficacy and safety. Throughout this translation pathway, organoids offer a human-relevant intermediate layer that can refine dose and regimen selection before trials and, during trials, help interrogate responder versus non-responder biology to guide iterative development.

Organoids can further unify formulation screening and patient stratification within a single experimentally tractable platform, thereby reducing uncertainty along the path from engineering design to clinical implementation. For example, multi-donor organoid biobanks can approximate real-world heterogeneity and prospectively identify subgroups most likely to benefit, enabling their deliberate enrichment in targeted trials. This paradigm has already gained traction in oncology for individualized regimen selection and is plausibly transferable to chronic conditions such as OSF, where interpatient variability and slow disease kinetics frequently obscure therapeutic signals (Tong, 2024).

In sum, this review advances a translational framework centered on quantifiable local exposure enabled by delivery systems and predictable, organoid-supported readouts, clarifying how EGCG’s multi-target anti-fibrotic activity can be moved from conceptual plausibility toward a more reproducible clinical trajectory. By leveraging nanotechnologies to improve stability and lesion-relevant exposure, deploying local biomaterials to enhance mucosal residence and patient adherence, and integrating organoids with biomarker strategies for precise stratification and response prediction, an EGCG-based, safe, and practical disease-modifying approach for OSF could realistically enter clinical testing in the foreseeable future. If successful, it would meaningfully alleviate the burden borne by the large population living with OSF and establish a template for therapeutic development in other oral fibrotic disorders. Future work should continue to close the evidentiary loop outlined here, iteratively strengthening each link, to translate this promising strategy into clinical reality.
